# Earlswood Asylum

**Published:** 1893-07-08

**Authors:** 


### FESTIVAL DINNERS AND MEETINGS.

### EARLSWOOD ASYLUM.

The annual festival dinner in aid of the funds of this asylum, took place on Tuesday night, June 27th, at Vintners' Hall, by kind permission of the Vintners' Company. Alderman J. V. Moore presided. After the usual toasts the Chairman proposed "The Earlswood Asylum, the Board of Management, the Stewards and Staff," and said it was his part that evening to plead for those who were unable to plead for themselves. At the present time there are in the institution at Redhill upwards of 600 patients, and the Commissioners in Lunacy report very favourably upon the care bestowed upon the inmates. These require very different treatment from the inmates of any other asylum, possibly from any other charitable institution. 199 of the male, and 41 of the female patients at the present moment are occupied in remunerative pursuits. Asylum is scarcely the word to apply to such an institution as this; it would be better described as a training school, a hospital, and a beautiful home. The toast was responded to by Mr. H. G. Hoare and Dr. L. Francis. Subscriptions amounting to £2,000 were announced. The total expenditure for the past year has reached the sum of nearly £15,000.

